# Plasmapheresis for the Treatment of Anti-M Alloimmunization in Pregnancy

**DOI:** 10.1155/2020/9283438

**Published:** 2020-02-07

**Authors:** Yohei Maki, Junko Ushijima, Seishi Furukawa, Hiroko Inagaki, Hiroyuki Takenouchi, Shouichi Fujimoto, Hiroshi Sameshima

**Affiliations:** ^1^Department of Obstetrics and Gynecology, Faculty of Medicine, University of Miyazaki, Miyazaki, Japan; ^2^Dialysis Division, University of Miyazaki Hospital, Miyazaki, Japan; ^3^Department of Transfusion and Cell Therapy, University of Miyazaki Hospital, Japan; ^4^Department of Hemovascular Medicine and Artificial Organs, Faculty of Medicine, University of Miyazaki, Miyazaki, Japan

## Abstract

Intrauterine transfusion is the standard antenatal treatment for a fetus with severe anemia. Plasmapheresis is an alternative treatment for cases with a history of severe hemolytic disease of the fetus and newborns at less than 20 weeks of gestation. There is only one previous report of plasmapheresis for the anti-M alloimmunization in pregnancy, and we report here on the successful treatment of plasmapheresis for anti-M alloimmunization. A woman with a history of intrauterine fetal death at 24 weeks of gestation due to severe fetal anemia caused by anti-M alloimmunization received plasmapheresis once or twice a week from 14 weeks of gestation onward. An intrauterine blood transfusion was conducted at 28 weeks, and a cesarean section was performed at 31 weeks. The infant had anemia and jaundice but was discharged at day 46. Plasmapheresis may delay the development of fetal anemia and reduce the risk of early and repeat intrauterine transfusion in cases of anti-M alloimmunization in pregnancy.

## 1. Introduction

Red cell alloimmunization in pregnancy causes hemolytic disease of the fetus and newborn (HDFN), resulting in fetal anemia, fetal hydrops, and fetal death. Intrauterine transfusion for the fetus with severe anemia is the standard antenatal treatment with survival rates of approximately 90-100% [1]. However, intrauterine transfusion is feasible after 20 weeks of gestation, with several case reports and case series showing the usefulness of alternative treatment, including plasmapheresis, intravenous immunoglobulin (IVIG), or a combination thereof for cases with a history of pregnancy with severe HDFN at less than 20 weeks of gestation. A guideline by the American Society for Apheresis published in 2016 proposes that plasmapheresis should be considered for these cases early in pregnancy and continued until intrauterine transfusion can safely be administered [2] despite the low evidence level. The major target antibody in the case reports and series is the anti-D antibody, which is the most frequent cause of HDFN.

Anti-M antibody can cause M/N incompatibility. A review reported the severity of anti-M alloimmunization varies from asymptomatic to fetal death [3], although a retrospective study involving 195 pregnancies showed the prevalence of severe HDFN caused by anti-M alloimmunization is extremely low [4]. There is only one report that showed the treatment with plasmapheresis for the anti-M alloimmunization in pregnancy succeeding in having a healthy infant [5]. Therefore, whether plasmapheresis is effective in cases with anti-M alloimmunization is unclear. We report here on the successful treatment using a new modality of simple plasma exchange (selective plasma exchange, SePE), for a case of anti-M alloimmunization with a history of fetal death due to HDFN in a previous pregnancy.

## 2. Case Presentation

The patient was a 36-year-old gravida 7, para 4 woman. The first three pregnancies with an ex-husband were uneventful, and the antibody screening was negative. She had two pregnancies with the present partner, but those resulted in artificial abortions. At the sixth pregnancy, the antibody screen was positive for anti-M with an IgG titer of 128. Antibody screening was performed in the microtube column agglutination techniques using IH-1000 (Bio-Rad Laboratories, Hercules, CA, USA), an automated blood screening system. Low-ionic strength solution-indirect antiglobulin techniques (LISS/IAT) and NaCl/enzyme test were used with a three-cell panel (ID-Diacell I-II-III, Diamed, Cressier, Switzerland) and ID-Dia (Diego) Positive (Bio-Rad Laboratories) for antibody screening and an 11-cell panel (ID-DiaPanel, Diamed) for subsequent antibody identification. The ultrasonography examination showed increased peak systolic velocity in the middle cerebral artery (MCA-PSV) of 1.5 multiples of median (MoM), suggesting fetal anemia at 19 weeks. Intrauterine infusion was unfeasible due to the unsuitable umbilical cord position. The fetus had hydrops fetalis at 24 weeks. Intraperitoneal transfusion was performed, but the pregnancy resulted in fetal death on the day of transfusion. At the seventh current pregnancy, the antibody test was positive for anti-M antibody with an IgG titer of 128 again at 13 weeks. Her blood type was NN and her husband's was MM, indicating the fetus' blood type was MN with a 100% chance of being at risk of HDFN. Selective plasma exchange (SePE) by using a selective membrane plasma separator (EVACURE EC-4A10, Kawasumi Laboratories Inc., Tokyo, Japan) was started twice a week from 14 weeks. For each procedure, 3000 mL plasma volume was replaced by 5% albumin. Since the titer of anti-M IgG was reduced to 32 at 16 weeks, SePE was performed once a week from 17 weeks onwards. The titer of anti-M IgG then became elevated at a level of 256 after 20 weeks, and thus, SePE was performed twice a week again from 23 weeks, and the replacement volume was increased to 3500 mL. IVIG of 500 mg/kg was replaced every three weeks since total IgG was reduced to approximately 150 mg/dL after serial SePE. A weekly ultrasound examination for MCA-PSV was performed from 18 weeks. At 28 weeks, MCA-PSV was increased to 1.6 MoM again. Percutaneous umbilical blood sampling was performed and fetal hematocrit was 20%. An intrauterine blood transfusion via the umbilical vein was done. The estimated hematocrit after transfusion was 35%. At 31 weeks, an intermittent sinusoidal pattern was observed, although ultrasound examination showed MCA-PSV of 1.27 MoM. We performed a cesarean section (Table 1). A male infant was live born weighing 1618 g with an Apgar score of 7 at 1 minute and 8 at 5 minutes. Umbilical artery blood gas analysis showed a pH of 7.39. Arterial hematocrit was 26%. His blood type test was MN as expected. The direct Coombs test was negative although the titer of anti-M IgG was 32. He needed phototherapy for the first 11 days and five transfusions of 10 ml/kg red blood cells. He was discharged at day 46 with normal brain findings on magnetic resonance imaging.

## 3. Discussion

Plasmapheresis removes IgG, including the red cell alloantibody in the maternal circulation, resulting in decreasing the amount of alloantibody transportation to the fetus. The survival rate of severe HDFN with plasmapheresis treatment is approximately 75% [2]. The majority of the past reports have consisted of the anti-D alloimmunization cases, but the present case suggests that plasmapheresis is also useful for anti-M alloimmunization. The direct Coombs test was negative in the infant indicating anti-M IgG titer was reduced to low level by plasmapheresis. However, anti-M alloimmunization is known to suppress red blood cell production at the progenitor cell level [3]. This could be the main cause of fetal anemia in this case.

There are several considerations regarding plasmapheresis for HDFN. First, there is no established protocol. The frequency of plasmapheresis varies among the reports from just three times at the first or second trimester [6] to three times a week throughout pregnancy [7]. The optimal volume of plasma processed is also not well known. The physiologic blood volume expansion during pregnancy should be considered as we increased the plasma processed after 23 weeks of gestation. Second, the antibody titer is not useful as a treatment marker in cases with anti-M alloimmunization. In cases of anti-D alloimmunization, the severity is higher with a higher titer. Therefore, the anti-D titer is a good marker for the treatment with plasmapheresis, especially before 18 weeks of gestation when the MCA-PSV is unreliable. Furthermore, the anti-M antibody titer is not correlated with the severity of HDFN [3]. The first case report of plasmapheresis for anti-M alloimmunization during pregnancy did not describe the anti-M antibody titer [4]. We increased the frequency of plasmapheresis in accordance with the titer, although the optimal titer is unclear. Last, IVIG has also been used for HDFN. The actual mechanism of IVIG is unclear, but some explanations have been proposed, including (1) the increase in the catabolism of IgG including harmful antibodies by the saturation of FcRn, which is abundant in endothelial cells and responsible for IgG catabolism, (2) the inhibition of the activation of the placental Fc receptors which leads to the blockade of antibody passage across the placenta, (3) the saturation of the Fc receptors in the fetal reticuloendothelial system which results in decreased phagocytosis of opsonized fetal cells, and (4) the inhibition of maternal antibody production by negative feedback [8–10]. A recent study reported that IVIG treatment alone succeeded in delaying the development of fetal anemia for 15 days compared to previous pregnancies [11]. The combination of IVIG and plasmapheresis is commonly performed in the case reports and series. Weekly high-dose IVIG of 1 g/kg maternal body weight is commonly administered [6, 11, 12]. The cost is a significant issue. Since maternal IVIG treatment for HDFN is not covered by public medical insurance in our country, we administered only 500 mg/kg IVIG every three weeks according to the label indication of hypogammaglobulinemia. Combination treatment should be considered if possible, since it may enhance the effect of plasmapheresis [1].

Plasmapheresis during pregnancy is thought to be safe [2]. No major complications occurred in our case. Spine position should be avoided to prevent hypotension, especially in later pregnancy. Large molecular coagulation factors can be decreased after repeated plasmapheresis with albumin replacement. We chose SePE, which removes small- and medium-sized molecules, including IgG without removing the large molecules such as fibrinogen and factor 13, and we confirmed the retention of these coagulation factors after every procedure. SePE can lower the risk of bleeding compared with other plasmapheresis modalities.

In conclusion, plasmapheresis may delay the development of fetal anemia and reduce the risk of early and repeat intrauterine transfusion in cases with anti-M alloimmunization in pregnancy.

## Figures and Tables

**Table 1:** Course of treatment of selective plasma exchange, anti-M titers, and the peak systolic velocity in the middle cerebral artery.

Gestational age	SePE	Anti-M antibody titer		MCA-PSV (MoM)	
		Pre SePE	Post SePE		
13 5/7		128			
14 4/7	1				SePE 2/week
15 0/7	2	512	128		
15 4/7	3				
16 0/7	4	64	32		SePE 1/week
17 0/7	5	64	32		
18 0/7	6	64	32	1.01	
19 0/7	7	64	32	0.97	
20 0/7	8	256	256	1.01	
21 0/7	9	256	256	0.86	
22 0/7	10	256	256	0.85	
23 1/7	11	128	64	0.95	SePE 2/week
23 5/7	12	256	128	1.03	
24 1/7	13	256	128		
24 3/7	14	128	64	1.01	
24 6/7	15	128	64		
25 4/7	16	64	64	1.27	
26 0/7	17	64	64	1.01	
26 4/7	18	128	64		
27 0/7	19	256	128		
27 4/7	20	128	32	1.56	
28 0/7	21	512	256	1.24	
28 4/7	22	256	256	1.6	
28 6/7				1.5	IUT
29 0/7	23	128	128	1.24	
29 4/7	24	256	64	1.3	
30 0/7	25	256	128	1.48	
31 0/7				1.27	Delivery

SePE: selective plasma exchange; MCA-PSV: the peak systolic velocity in the middle cerebral artery; MoM: multiples of median; IUT: intrauterine blood transfusion.
